# Abnormal DLPFC activation in severe methamphetamine use disorder during abstinence: fNIRS evidence from Stroop task

**DOI:** 10.3389/fpsyt.2026.1863806

**Published:** 2026-08-05

**Authors:** Huiting Luo, Dan Luo, Qiao Tang, Jiaxi Zhang, Jiajun Xu, Jing Li

**Affiliations:** Mental Health Center, West China Hospital of Sichuan University, Chengdu, China

**Keywords:** abstinence, brain function, fNIRS (functional near infrared spectroscopy), inhibitory control, methamphetamine

## Abstract

**Introduction:**

Methamphetamine use disorder (MUD) is a chronic relapsing disorder characterized by impaired inhibitory control, particularly in severe cases. However, the neural mechanisms underlying cognitive control during abstinence remain unclear.

**Methods:**

This study employed functional near-infrared spectroscopy (fNIRS) and the Stroop task to examine brain activation and behavior in 73 abstinent individuals with severe MUD (SMUDs) and 51 matched healthy controls (HCs). Independent-samples t-tests assessed group differences, with false discovery rate correction across fNIRS channels. Covariate-adjusted sensitivity analyses controlled for education and Alcohol Use Disorders Identification Test scores. Moderation analysis examined whether abstinence duration modified activation–behavior associations. Pearson correlations and Fisher’s r-to-z tests examined direct and subgroup associations.

**Results:**

SMUDs showed reduced activation in channels 32 and 43 (CH32 and CH43) within the left dorsolateral prefrontal cortex (DLPFC) compared with HCs, despite no significant group differences in conventional Stroop behavioral indices. These activation differences remained significant after covariate adjustment. Abstinence duration significantly moderated the association between CH43 activation and the reaction-time difference score (DRT; incongruent RT minus congruent RT), whereas abstinence duration was not directly correlated with CH43 activation. The positive association between CH43 activation and DRT was significant at shorter abstinence durations but not at longer abstinence durations.

**Discussion:**

These findings reveal left DLPFC hypoactivation and altered neural–behavioral coupling in SMUDs. The observed DLPFC hypoactivation remained robust after adjustment for education and AUDIT scores, consistent with persistent SMUD-related prefrontal dysfunction. Moreover, early abstinence may be characterized by altered or less efficient activation–behavior coupling, highlighting this stage as a potential window for targeted neuromodulatory interventions aimed at supporting cognitive recovery.

## 1 Introduction

Methamphetamine use disorder (MUD) poses a severe global public health issue, contributing to both individual health deterioration and increased socioeconomic burden. According to the United Nations Office on Drugs and Crime (UNODC) World Drug Report 2023, methamphetamine (METH) related abuse constitutes over 50% of all drug abuse cases in East and Southeast Asia (1). In China, data from the 2022 Drug Situation Report indicate that approximately 588,000 individuals use METH, comprising 52.3% of the national drug-using population (2). As a potent central nervous system stimulant, long-term METH use leads to considerable neurotoxicity, cognitive impairment, and social dysfunction, thereby significantly contributing to the global disease burden (3). These observations underscore the need to elucidate the neurobiological mechanisms underlying MUD to inform targeted intervention strategies.

The severity of MUD is closely related to its impairments of cognitive functions, with deficits in inhibitory control considered a central neuropsychological mechanism driving addiction (3, 4). Inhibitory control refers to an individual’s ability to suppress impulsive behaviors and regulate attention to achieve goal-directed actions. Impairment in this function leads to compulsive drug-seeking and elevated relapse risk in individuals with substance use disorder (5, 6). Chronic METH use disrupts the structure and function of prefrontal-striatal circuits and alters dopaminergic neurotransmission, particularly by impairing dopamine release and receptor sensitivity in the brain’s reward system. These changes disrupt the functional balance between reward and cognitive control networks, particularly impacting the dorsolateral prefrontal cortex (DLPFC) (7). Resulting deficits in executive function manifest as impaired decision-making, reduced cognitive flexibility, diminished working memory, and increased impulsivity (8). However, the dynamic changes in DLPFC function across different stages of METH abstinence in MUD patients remain inadequately explored, limiting more comprehensive understanding of the addiction mechanisms. This knowledge gap compels further examination of the progress and limitations of existing research.

Neuroimaging studies have been instrumental in providing the neurobehavioral characteristics associated with MUD. Prior studies have shown that METH users exhibit atypical patterns of prefrontal activation during cognitive tasks (9). Specifically, several studies have reported significantly diminished activation of the dorsolateral prefrontal cortex (DLPFC) in MUD patients during tasks requiring cognitive control, such as the color-word Stroop task, which suggests deficits in inhibitory control and conflict monitoring (10, 11). However, the results of these studies are not uniform: individuals in the early stages of abstinence may show compensatory hyperactivation (12), while those in long-term abstinence might exhibit cognitive recovery, albeit with reduced activation (13) or increased gray matter volume (14). Additionally, studies using emotional conflict tasks have found that MUD patients might activate the right ventrolateral prefrontal cortex (VLPFC) in specific contexts, indicating the involvement of prefrontal regions in cognitive and emotional regulation (15). These inconsistencies may arise from variations in abstinence duration, the severity of MUD, task designs, and individual differences such as age, sex, and comorbid conditions, which all influence neural function. Despite the evidence provided by these studies (16, 17), there remains a lack of longitudinal research tracking continuous changes in neural functions across different abstinence times. More critically, research focusing on the neurobehavioral characteristics of severe MUD patients (defined as exhibiting six or more symptoms according to DSM-5) is still insufficient (18).

The shortcomings in existing research point to the crucial role of abstinence duration and the severity of MUD in shaping neurobehavioral characteristics. Abstinence duration significantly influences prefrontal function and inhibitory control performance (19). Studies suggest that early abstinence stages may be characterized by compensatory neural activation that leads to inefficient processing, while long-term abstinence may be associated with functional recovery (20), though this dynamic trajectory has yet to be systematically validated. Additionally, patients with severe MUD (SMUD), owing to their longer drug-use history and more pronounced cognitive impairments, exhibit more persistent deficits in neural function and a higher risk of relapse (21, 22). However, existing research has seldom explored how abstinence duration in SMUD patients affects neurobehavioral characteristics, which could have important implications for personalized treatment strategies. Moreover, the portable functional near-infrared spectroscopy (fNIRS) technology, which offers real-time and ecologically valid assessments (23), provides new opportunities for MUD research, yet its application remains underutilized. This technology’s potential to provide more authentic neural behavioral data could help uncover the cortical activation patterns of SMUD patients at different stages of abstinence. Based on this, developing precise physical treatment plans tailored to the severity and abstinence duration of MUD patients could be an urgent issue to address prior to implementing targeted interventions.

Based on the current state of research, we hypothesize that the relationship between DLPFC activation and cognitive control performance may vary according to the stage of abstinence. Therefore, this study recruited individuals with SMUD (SMUDs) and healthy controls (HCs) to complete a color-word Stroop task while undergoing measurement with portable fNIRS. This study aims to investigate the differences of brain activation and behavioral performance between SMUDs and HCs, and further to explore the relationships among abstinence duration, brain activation, and behavioral performance in SMUDs. By uncovering the neurobehavioral characteristics of SMUDs at different stages of abstinence, this study will provide new insights into neurobehavioral features in SMUD and lay a scientific foundation for personalized intervention strategies based on the abstinence duration.

## 2 Methods

### 2.1 Patients

Male SMUDs were recruited between July and December 2023 from the Chengdu Compulsory Detoxification Center, Sichuan Province, China. All participants tested positive for METH on urine toxicology screening upon admission. They underwent structured psychiatric interviews by a licensed psychiatrist, documenting their drug use history and psychiatric symptoms. Participants also completed a psychiatric evaluation, a case report form (CRF), and fNIRS brain imaging.

Inclusion criteria for the SMUD group were as follows: aged 18-55; right-handed; Han Chinese ethnicity; primary school education or higher; able to complete tasks; met DSM-5 criteria for severe methamphetamine use disorder (≥6 criteria); able to complete neuropsychological assessments; provided informed consent. Exclusion criteria encompassed: diagnosis of major psychiatric disorders (e.g., schizophrenia, bipolar disorder); positive family history of psychiatric illness; regular use of other illicit substances (excluding tobacco and alcohol); history of serious medical conditions (e.g., multiple sclerosis, liver cirrhosis, malignancies); history of significant head trauma or neurosurgical procedures; confirmed HIV or syphilis infection; and presence of color vision deficiency.

HCs were recruited through local advertisements near the Mental Health Center, West China Hospital of Sichuan University and were matched to the SMUD group based on gender, age, and ethnicity. Eligibility criteria for HCs included: age 18–55 years; right-handed Han; Chinese ethnicity; no current or past psychiatric disorders, as confirmed by the Mini International Neuropsychiatric Interview (MINI, version 7.0); absence of psychoactive substance use (excluding tobacco and alcohol); and ability to provide written informed consent. Exclusion criteria were consistent with those applied to the SMUD group.

The study protocol and informed consent procedures complied with the principles outlined in the Declaration of Helsinki and were approved by the Biomedical Ethics Committee of West China Hospital, Sichuan University (Ethics Approval No. 2022 Review [847]). All participants were fully informed about the study procedures and provided written informed consent prior to participation.

### 2.2 Data collection

#### 2.2.1 Demographic and substance use assessment

A custom-designed CRF was used to collect detailed information from all participants with SMUD. General demographic data included age, ethnicity, and years of education. Core characteristics of METH use were also recorded, including age at first METH use, duration of continuous METH use, dosage and frequency during initial and regular use periods, history of maximum dose and highest frequency of use, and duration of current abstinence.

#### 2.2.2 Visual analog scale for craving

The VASc was used to assess participants’ subjective craving for drug following exposure to METH-related cues (24). Participants were presented with a 10 cm horizontal line, anchored with “0” on the left (no craving) and “10” on the right (extreme craving). Higher scores indicated greater psychological craving for METH. Participants were instructed to mark a point on the line that best reflected the intensity of their current craving at that moment.

#### 2.2.3 Alcohol use disorders identification test

The AUDIT is a widely used screening tool for identifying hazardous and harmful patterns of alcohol consumption (25). Total scores are interpreted as follows: low-risk drinking (<8 points), hazardous drinking (8–15 points), harmful drinking (16–19 points), and probable alcohol dependence (≥20 points).

#### 2.2.4 Fagerström test for nicotine dependence

The FTND is a standardized instrument used globally to assess nicotine dependence and tobacco use patterns (26). The scale consists of six items with a total score ranging from 0 to 10. A score of ≥6 is generally considered indicative of high nicotine dependence.

#### 2.2.5 Beck depression inventory

The BDI is a widely used self-report instrument for assessing depressive symptoms, with well-established reliability and validity. In the present study, we used the 13-item short version (27), which evaluates symptoms experienced over the past week. Each item is rated on a 4-point Likert scale ranging from 0 (absent) to 3 (severe). Total scores are categorized as follows: no depression (0–4), mild depression (5–7), moderate depression (8–15), and severe depression (≥16) (28).

#### 2.2.6 Beck anxiety inventory

The BAI is a 21-item self-report measure designed to assess subjective anxiety symptoms (29). Each item is rated on a scale from 0 (not at all) to 3 (severely—it bothered me a lot), yielding a total score ranging from 0 to 63. Scores are interpreted as follows: minimal anxiety (0–7), mild anxiety (8–15), moderate anxiety (16–25), and severe anxiety (26–63).

#### 2.2.7 Pittsburgh sleep quality index

The Pittsburgh Sleep Quality Index (PSQI) is a self-report instrument used to assess sleep quality over the past month (30). The total score ranges from 0 to 21, with higher scores indicating poorer sleep quality. A total score >5 is generally considered indicative of poor sleep quality.

### 2.3 fNIRS data acquisition

Subjects were comfortably seated in a quiet room and a multichannel near-infrared optical imaging system (NirScan, Danyang Huichuang Medical Equipment Co, Ltd, China) to measure hemoglobin concentration. The sampling frequency was 11 Hz. The fNIRS system utilized two primary wavelengths: 850 nm and 730 nm, with an isosbestic reference wavelength calibrated at 808 nm. A total of 31 optodes were placed on the scalp, comprising 15 light emitters and 16 detectors, with an inter-optode distance fixed at 3 cm. The center of the optode array was aligned with the FPz position according to the international 10–20 system. The array covered bilateral prefrontal cortex (PFC) and temporal cortical regions. The lowest row of optodes was aligned along the Fp1-Fp2 axis. The probes were arranged in a 3 × 11 matrix, forming a total of 48 measurement channels.

### 2.4 Data analysis of fNIRS

The NirSpark software (Huichuang, China) was used to analyze the fNIRS data (31). For preprocessing, a band-pass filter with a cutoff of 0.01-0.1 Hz was used to eliminate physiological noise (e.g., breathing, cardiac activity) (32). Optical density was converted to changes in oxyhemoglobin (HbO) concentration using a modified Beer-Lambert law. The 5 seconds before each block of the Stroop task was selected as the pre-task baseline range, and the 10 seconds after each block was selected as the post-task baseline range. A linear fit was applied to the data between the two baselines, and the task period of 31.8 seconds in the middle was used as the time window to calculate the relative change in the average oxygenated hemoglobin concentration during each block. The total average of the HbO activation change values of all 48 channels of all subjects in the five blocks was calculated.

### 2.5 Stroop task

**Figure 1:**
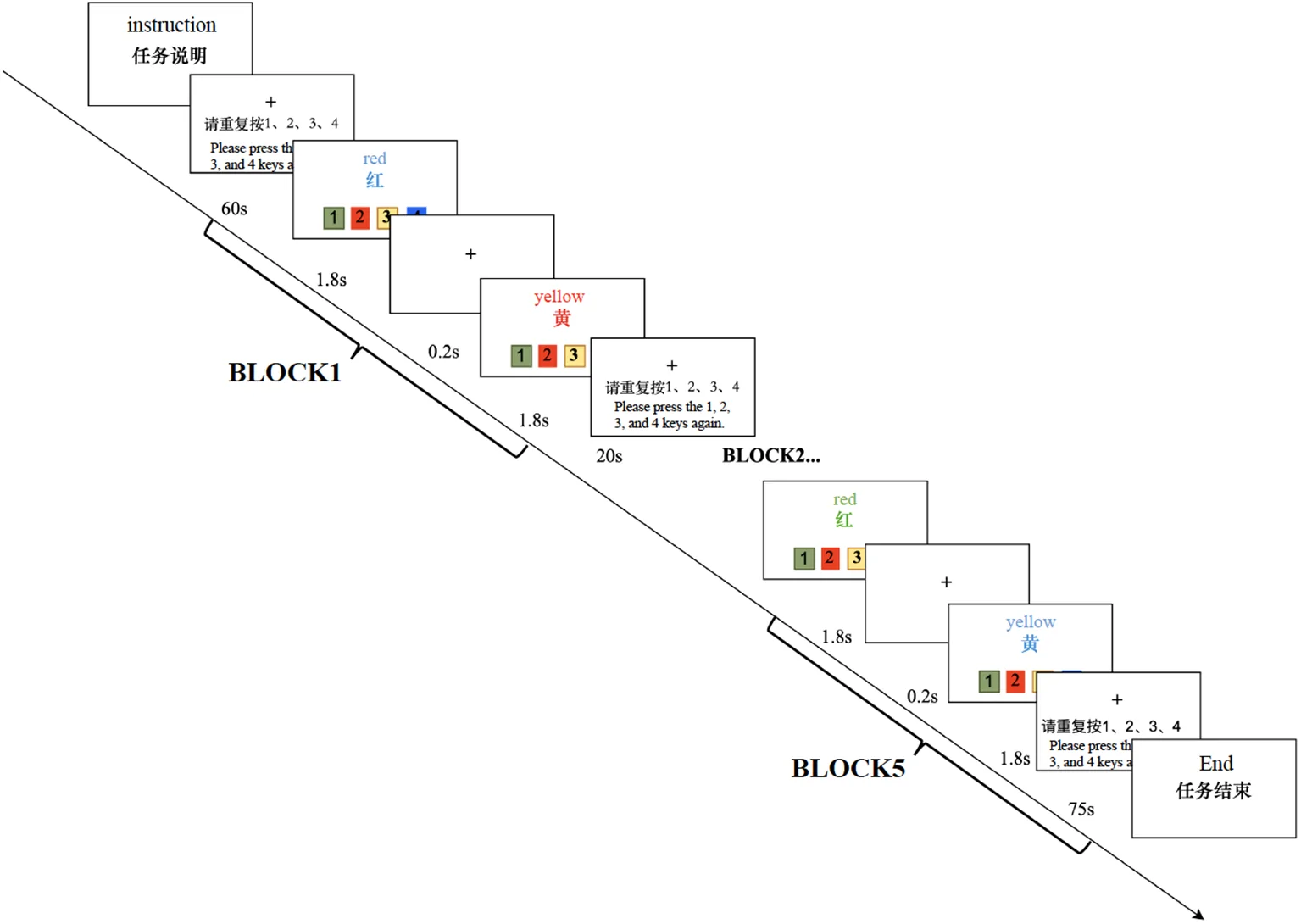
Design of the Stroop task. The task consisted of five repeated Stroop task blocks with identical trial composition, stimulus materials, and experimental parameters. Each block contained 16 trials, including 4 congruent and 12 incongruent trials. The congruent trials consisted of four color-word matched stimuli corresponding to red, yellow, blue, and green, whereas the incongruent trials covered all 12 possible color-word mismatched combinations generated from the four colors. Trials within each block were pseudo-randomized using sampling without replacement before reset, and the trial sequence differed across blocks. Each stimulus was presented for 1800 ms, with a 200-ms fixation cross displayed between trials. Fixation and sequential key-pressing baseline/control periods were administered before Block 1, between adjacent blocks, and after Block 5.

The Stroop task is a classic paradigm used to assess cognitive control processes (33). It involves selective attention and inhibitory control, which are key components of executive function (34). In this study, the Stroop task was programmed and administered using E-Prime 3.0 software. The overall task sequence is shown in **Figure 1**.

A block-designed Stroop color-word task was adopted. The task consisted of five repeated task blocks with identical trial composition, stimulus materials, and experimental parameters. Repeated block acquisition was used to improve the signal-to-noise ratio of the fNIRS hemodynamic signals. Each block contained 16 trials, including 4 congruent trials and 12 incongruent trials. In the congruent trials, the meaning of the Chinese color word was consistent with its ink color. These trials consisted of four color-word matched stimuli, corresponding to red, yellow, blue, and green. In the incongruent trials, the meaning of the Chinese color word was inconsistent with its ink color. The incongruent trials covered all 12 possible color-word mismatched combinations generated from the four colors. Therefore, the four colors appeared with balanced frequencies across the full set of trials. Within each block, trials were presented using pseudo-random sampling without replacement before reset, according to the “No Repeat After Reset” rule in E-Prime. Congruent and incongruent trials were randomly interspersed within each block. The trial presentation sequence differed across the five task blocks, which helped prevent participants from forming stimulus expectations or developing order-learning effects.

Participants’ reaction time (RT) and response accuracy were recorded for each trial. In each trial, a Chinese character was displayed in a color that was either congruent or incongruent with its meaning. Below the stimulus, four color options and their corresponding response keys were presented. Based on prior studies (35) and previous research by our team, each stimulus was presented for 1800 ms. A fixation cross (“+”) was displayed between trials for 200 ms. Following each of Blocks 1-4, participants were instructed to fixate on the central cross and repeatedly press keys 1 through 4 for 20 seconds, at a pace of approximately one key press per second. Additionally, a similar fixation and key-pressing task was performed for 60 seconds prior to Block 1 and 75 seconds after Block 5. The total task duration was 374 seconds. All participants completed a practice session before the formal test to ensure full understanding of the task requirements.

### 2.6 Statistical analysis

Statistical analyses were performed using IBM SPSS Statistics for Mac (version 26.0) and the PROCESS macro for SPSS (version 4.2). Continuous variables are presented as mean ± standard deviation (SD). The difference in reaction time DRT was calculated within each participant as incongruent-condition RT minus congruent-condition RT (incoRT − coRT), consistent with the operationalization used in previous Stroop research (36). Independent-samples t-tests were used to compare demographic characteristics, clinical variables, behavioral measures, and HbO activation values across the 48 fNIRS channels between the SMUD and HC groups, as well as between SMUD subgroups stratified by abstinence duration. Clinical variables included substance-use-related measures and psychiatric symptom assessments, including AUDIT, FTND, BDI, BAI, and PSQI. METH-related characteristics, including age at first METH use, duration of METH use, METH dose and frequency, and abstinence duration, were summarized descriptively and compared between SMUD subgroups when applicable. Pearson correlation analysis was used to examine the association between abstinence duration and CH43 activation across all SMUD participants and the association between CH43 activation and DRT within the STW and LTW subgroups. Fisher’s r-to-z transformation was used to compare the subgroup correlation coefficients. Statistical significance was set at a two-tailed p < 0.05. To control for multiple comparisons across fNIRS channels, the false discovery rate (FDR) correction was applied.

Given the significant between-group differences in years of education and AUDIT scores, additional covariate-adjusted sensitivity analyses were conducted to evaluate the robustness of the primary fNIRS findings. Analysis of covariance (ANCOVA) was performed for the channels that remained significant after FDR correction, with group as the between-subject factor, HbO activation as the dependent variable, and years of education and AUDIT scores as covariates. The adjusted group effects were reported using F statistics, p values, and partial eta-squared (η²p) effect sizes.

To examine whether abstinence duration moderated the relationship between prefrontal activation and behavioral performance, moderation analyses were conducted using the PROCESS macro. Behavioral measures were entered as dependent variables, abstinence duration was specified as the moderator, and HbO activation in channels showing significant group differences between the SMUD and HC groups was entered as the independent variable. Age and years of education were included as covariates in all models. When significant interaction effects were identified, simple slope analyses were performed to facilitate interpretation of the moderation effects.

For the stepwise regression analyses, independent variables included age, years of education, clinical characteristics that differed significantly between SMUD subgroups, and HbO activation in channels showing significant group differences between the SMUD and HC groups. Behavioral measures exhibiting significant subgroup differences were entered as dependent variables. A stepwise regression procedure combining forward selection and backward elimination was used to identify significant predictors. Multicollinearity was assessed using variance inflation factors (VIFs), with values greater than 5 indicating potential multicollinearity. Autocorrelation of residuals was evaluated using the Durbin–Watson statistic, with values close to 2 indicating independence of errors.

## 3 Result

### 3.1 Demographic and clinical data

A total of 73 SMUDs and 51 HCs were recruited for this study. Compared to the SMUDs, HCs had significantly more years of education (14.12 ± 3.73 vs. 9.44 ± 3.04, p < 0.05) and lower scores on the AUDIT (2.71 ± 5.90 vs. 5.26 ± 7.73, p < 0.05). No significant group differences were observed in age, FTND scores, or PSQI scores. Detailed METH use patterns and clinical characteristics of the SMUD group are presented in **Table 1**.

**Table 1:** Demographic and clinical information between SMUD and HC groups.

Variables	SMUD (n = 73)	HC (n = 51)	t	p value
Age (year)	34.55 ± 7.14	33.59 ± 9.39	0.646	0.519
Education (year)	9.44 ± 3.04	14.12 ± 3.73	-7.676	<0.001
FTND	4.05 ± 1.46	3.24 ± 2.67	1.996	0.050
AUDIT	5.26 ± 7.73	2.71 ± 5.90	2.054	0.042
Duration of METH use (month)	73.53 ± 51.64	NA	NA	NA
Dose at the beginning of use (g)	0.29 ± 0.37	NA	NA	NA
Frequency of use at the beginning (times/day)	0.34 ± 0.38	NA	NA	NA
Dose during regular use (g)	0.57 ± 0.48	NA	NA	NA
Frequency during regular use (times/day)	1.03 ± 1.19	NA	NA	NA
Highest dose of METH used in the past (g)	1.02 ± 0.81	NA	NA	NA
Highest frequency of METH used in the past (times/day)	1.83 ± 2.72	NA	NA	NA
METH abstinence duration (month)	15.92 ± 7.51	NA	NA	NA
DSM-5 diagnostic items	8.32 ± 1.68	NA	NA	NA
VASc	3.08 ± 3.13	NA	NA	NA
BDI	6.62 ± 4.36	NA	NA	NA
BAI	6.04 ± 7.31	NA	NA	NA
PSQI	4.85 ± 2.81	NA	NA	NA

SMUD, Severe Methamphetamine Use Disorder; HC, Healthy Controls; FTND, Fagerström Test for Nicotine Dependence; AUDIT, Alcohol Use Disorders Identification Test; VASc, Visual Analog Scale for Craving; BDI, Beck Depression Inventory; BAI, Beck Anxiety Inventory; PSQI, Pittsburgh Sleep Quality Index; NA, not applicable.

### 3.2 Group differences in the activation of HbO

**Figure 2:**
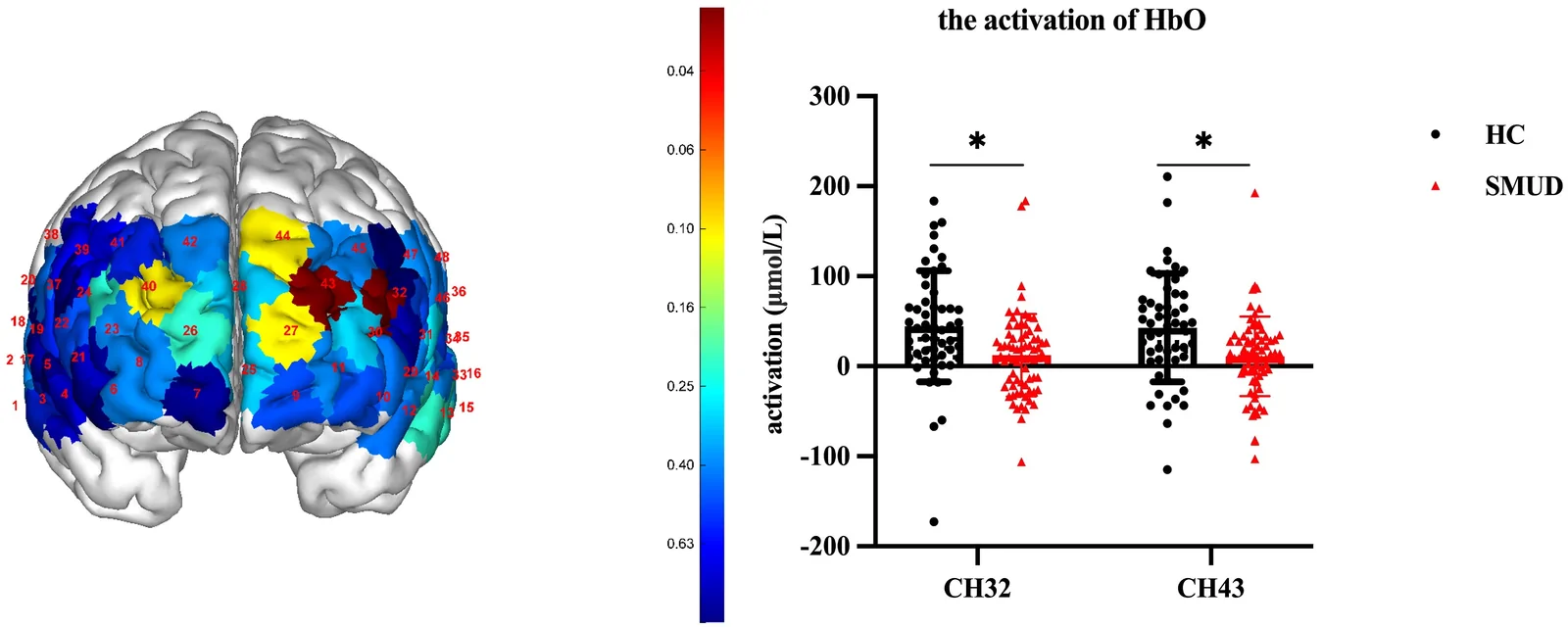
The activation of HbO during the Stroop task between SMUD and HC groups. SMUD, Severe Methamphetamine Use Disorder; HC, Healthy Control; CH32/43, HbO activation; *p < 0.05.

As shown in **Figure 2**, SMUDs exhibited significantly reduced HbO activation in channel 32 and channel 43 (CH32 and CH43), both located in the left DLPFC (Brodmann area 9), compared to HCs. These group differences in HbO activation were statistically significant after FDR correction, with adjusted p-values < 0.05 for both CH32 and CH43.

To further address the potential confounding effects of years of education and AUDIT scores, we conducted covariate-adjusted sensitivity analyses for the channels that survived FDR correction in the original channel-wise analysis. The group differences in CH32 (F = 18.2142, p < 0.0001, partial η² = 0.1347) and CH43(F = 13.9294, p = 0.0003, partial η² = 0.1064) remained significant after controlling for these covariates. Detailed results are presented in **Supplementary Table 1**.

### 3.3 Behavioral performance results

The analysis of the data depicted in **Table 2** reveals that there are no statistically significant differences between the SMUD and HC groups in terms of accuracy under the incongruent condition (incoACC), reaction time under the incongruent condition (incoRT), accuracy under the congruent condition (coACC), reaction time under the congruent condition (coRT), or the difference of reaction time (DRT) between the two conditions (all p > 0.05).

**Table 2:** Comparison of Stroop behavioral performance between SMUD and HC groups.

Variables	SMUD (n = 73)	HC (n = 51)	t	p value
incoACC (%)	73.15 ± 30.61	80.90 ± 26.86	-1.457	0.148
incoRT (ms)	935.56 ± 178.58	924.42 ± 172.27	0.347	0.729
coACC (%)	93.11 ± 8.75	93.27 ± 12.18	-0.088	0.930
coRT (ms)	866.76 ± 179.16	842.86 ± 172.04	0.743	0.459
DRT (ms)	105.21 ± 72.11	88.95 ± 66.85	1.213	0.228

SMUD, Severe Methamphetamine Use Disorder; HC, Healthy Control; incoACC, accuracy under the incongruent condition; incoRT, reaction time under the incongruent condition; coACC, accuracy under the congruent condition; coRT, reaction time under the congruent condition; DRT, the difference of reaction time.

### 3.4 The moderating effect of abstinence duration

As shown in **Table 3**, a significant interaction emerged between CH43 activation and METH abstinence duration in predicting DRT (p = 0.013), indicating that the association between CH43 activation and DRT varied as a function of abstinence duration. In contrast, the direct Pearson correlation between abstinence duration and CH43 activation was not significant (r = 0.091, p = 0.442), indicating that the moderation effect did not reflect a simple linear change in CH43 activation with abstinence duration. No significant interaction was observed for CH32 or for other behavioral indices (p > 0.05).

**Table 3:** To assess the moderating effect of abstinence duration on the relationship between brain activation and the Stroop task.

Variables	Model 1				Model 2				Model 3			
	B	SE	t	p	B	SE	t	p	B	SE	t	p
Age (year)	0.72	1.32	0.545	0.588	0.98	1.32	0.739	0.463	1.00	1.26	0.798	0.428
Education (year)	-1.04	3.37	-0.309	0.758	-1.41	3.35	-0.422	0.675	-0.01	3.23	-0.003	0.998
CH43 (mmol/L)	184.61	255.96	0.721	0.474	205.67	253.94	0.810	0.422	443.39	258.52	1.715	0.092
METH abstinence duration (month)					-1.84	1.28	-1.437	0.156	-2.05	1.22	-1.679	0.099
CH43* METH duration of abstinence									-91.475	35.42	-2.583	0.013
R2	0.017				0.053				0.159			
△F	0.318				2.066				6.670			
p	0.812				0.156				0.013			

Both the independent variable (CH43) and the moderator (duration of abstinence) were standardized prior to analysis. In Model 1, CH43 and two covariates (age and education) were entered. Model 2 added the moderator variable (duration of abstinence) to Model 1. Model 3 further included the interaction term to test the moderation effect.

**Figure 3:**
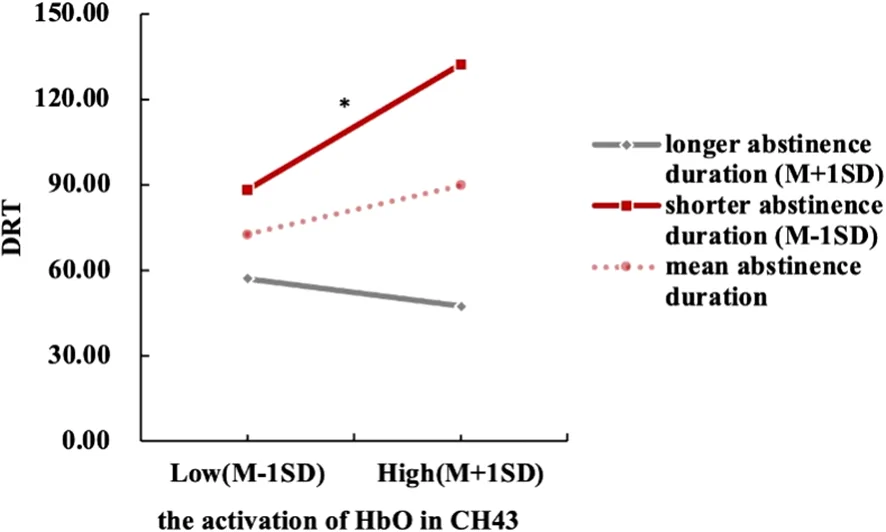
Simple slopes for the interaction between the activation and task performance. DRT, the difference of reaction time; CH43, HbO activation; *p < 0.05.

To further clarify the underlying pattern of the moderation effect, a simple slopes analysis was performed. As depicted in **Figure 3**, the association between HbO activation in CH43 and DRT varied significantly across levels of abstinence duration. Among individuals with shorter abstinence duration (M-1 SD), CH43 activation was significantly and positively associated with DRT (B = 1137.10, SE = 434.09, p = 0.011). At the mean level of abstinence duration, the positive association was attenuated and only marginally significant (B = 443.38, SE = 258.51, p = 0.092). In contrast, for those with longer abstinence (M + 1 SD), the relationship became negative, although it did not reach statistical significance (B = -250.33, SE = 299.23, p = 0.407).

### 3.5 Group differences in demographic, clinical, task performance and activation of HbO between subgroups

Based on the mean METH abstinence duration (month) (15.92 months), SMUDs were divided into two subgroups: a short-term abstinence (STW) subgroup with an average abstinence duration of 8.96 ± 3.61 months, and a long-term abstinence (LTW) subgroup with an average of 22.24 ± 3.16 months. As shown in **Table 4**, the STW subgroup reported a lower highest frequency of METH use in the past (1.13 ± 1.15 vs. 2.50 ± 3.52 times/day, p < 0.05) but higher AUDIT scores (8.11 ± 7.46 vs. 2.63 ± 7.09, p < 0.01) relative to the LTW subgroup. No significant differences were observed between the subgroups in age, education, other METH use characteristics, or scores on the BDI, BAI, and PSQI (all p > 0.05).

**Table 4:** Demographic, clinical, and task performance comparisons between subgroups.

Variables	STW (n = 35)	LTW (n = 38)	t	p value
Age (year)	33.49 ± 6.17	35.53 ± 7.89	-1.224	0.225
Education (year)	10.11 ± 2.72	8.82 ± 3.21	1.856	0.068
Duration of METH use (month)	81.11 ± 50.61	66.55 ± 52.26	1.207	0.231
Dose at the beginning of use (g)	0.34 ± 0.50	0.24 ± 0.20	1.133	0.261
Frequency of use at the beginning (times/day)	0.34 ± 0.38	0.34 ± 0.37	-0.041	0.967
Dose during regular use (g)	0.65 ± 0.63	0.49 ± 0.27	1.409	0.163
Frequency during regular use (times/day)	0.82 ± 0.81	1.21 ± 1.44	-1.399	0.166
Highest dose of METH used in the past (g)	1.10 ± 1.00	0.96 ± 0.59	0.745	0.459
Highest frequency of METH used in the past (times/day)	1.13 ± 1.15	2.50 ± 3.52	-2.244	0.030
VASc	3.77 ± 2.95	2.45 ± 3.19	1.838	0.070
FTND	4.34 ± 1.37	3.79 ± 1.51	1.635	0.106
AUDIT	8.11 ± 7.46	2.63 ± 7.09	3.213	0.002
BDI	6.66 ± 5.30	6.58 ± 3.34	0.075	0.941
BAI	7.49 ± 8.15	4.71 ± 6.26	1.639	0.106
PSQI	5.09 ± 2.93	4.63 ± 2.72	0.687	0.494
incoACC (%)	67.19 ± 36.14	78.65 ± 23.64	-1.590	0.117
incoRT (ms)	906.99 ± 190.34	974.66 ± 165.40	-1.615	0.111
coACC (%)	93.46 ± 8.26	92.78 ± 9.27	0.332	0.741
coRT (ms)	802.34 ± 173.57	918.59 ± 159.70	-2.980	0.004
DRT (ms)	124.33 ± 81.49	84.05 ± 53.94	2.213	0.031

STW, short-term abstinence; LTW, long-term abstinence; FTND, Fagerström Test for Nicotine Dependence; AUDIT, Alcohol Use Disorders Identification Test; VASc, Visual Analog Scale for Craving; BDI, Beck Depression Inventory; BAI, Beck Anxiety Inventory; PSQI, Pittsburgh Sleep Quality Index; incoACC, accuracy under the incongruent condition; incoRT, reaction time under the incongruent condition; coACC, accuracy under the congruent condition; coRT, reaction time under the congruent condition; DRT, the difference of reaction time.

**Figure 4:**
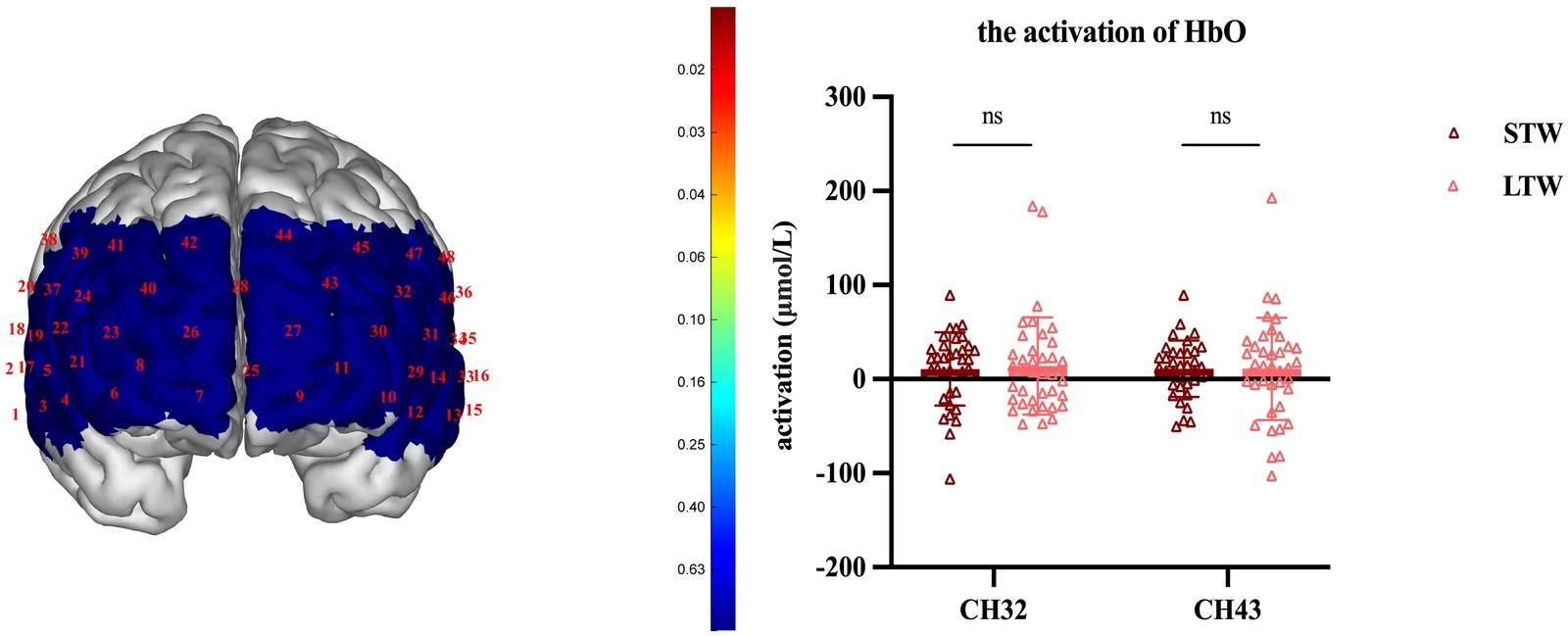
The activation of HbO during the Stroop task between STW and LTW. STW, short-term abstinence; LTW, long-term abstinence; CH32/43, HbO activation; ns, no significant.

Behaviorally, the STW subgroup exhibited shorter coRT (802.34 ± 173.57 ms vs. 918.59 ± 159.70 ms, p < 0.01) and a longer DRT (124.33 ± 81.49 ms vs. 84.05 ± 53.94 ms, p < 0.05). Because incoRT and accuracy did not differ significantly between the subgroups, the longer DRT should be interpreted cautiously as a larger RT difference score rather than unequivocal evidence of poorer inhibitory control. However, no significant subgroup differences were observed in HbO activation in CH32 or CH43 (**Figure 4**).

### 3.6 Regression analysis in the STW Group

Within the STW subgroup, CH43 activation was significantly positively correlated with DRT (r = 0.423, p = 0.011, n = 35), whereas this correlation was not significant in the LTW subgroup (r = −0.017, p = 0.918, n = 38). The difference between the two correlation coefficients was marginal but did not reach the conventional significance threshold (Fisher z = 1.915, p = 0.055). In the STW subgroup, stepwise regression further showed that age and CH43 activation jointly accounted for 30.2% of the variance in DRT (R² = 0.302, F = 6.056, p = 0.007; **Table 5**). All VIF values were below 5, indicating no evidence of multicollinearity among variables. The D-W statistic was approximately 2, suggesting the absence of autocorrelation among the residuals.

**Table 5:** Stepwise regression identifying variables associated with DRT in the STW group.

Variables	Unstandardized coefficient		Standardized coefficient	t	p value	Collinearity diagnostics	
	B	SE	Beta			VIF	Tolerance
Age (year)	4.42	1.98	0.35	2.233	0.034	1.005	0.995
CH43 (mmol/L)	1064.73	424.85	0.40	2.506	0.018	1.005	0.995
R2	0.302						
F	6.056						
P	0.007						
D-W	2.359						

DRT, the difference of reaction time; CH43, HbO activation in channel 43 (left DLPFC); SE, standard error; VIF, variance inflation factor; Tolerance = 1/VIF; D-W, the Durbin-Watson.

## 4 Discussion

This study utilized fNIRS to investigate prefrontal activation in SMUDs during the Stroop task and its relationships with behavioral performances. Despite similar behavioral performance, SMUDs showed reduced activation in the left DLPFC (CH32, CH43), indicating impaired prefrontal function. Moderation analysis revealed that METH abstinence duration significantly influenced the association between CH43 activation and DRT, an RT-based Stroop interference index. Specifically, a stronger positive coupling was observed in SMUDs with shorter abstinence durations. Consequently, a subgroup comparison was conducted by stratifying the SMUDs based on their abstinence durations. Within the STW subgroup, a stepwise regression analysis was performed. The final model indicated that the combined contribution of CH43 activation and age accounted for 30.2% of the variance in DRT. In contrast, no significant model was identified in the LTW subgroup, consistent with the lack of a significant association observed in the moderation analysis. These findings underscore abstinence duration as a critical factor in neural-behavioral coupling in abstinent SMUDs.

### 4.1 Reduced activation in DLPFC

The reduced left DLPFC activation observed in SMUDs aligns with prior studies (9, 11, 37) reporting structural (21, 38), metabolic (39), and functional (40, 41) abnormalities in the DLPFC among METH users. As a critical region for executive functioning and inhibitory control, the DLPFC supports the maintenance of task goals and the suppression of task-irrelevant information (42). Dysfunction in this region may therefore compromise cognitive-control processes that are central to addiction-related impairment.

An important finding of the present study is that SMUDs showed significantly reduced left DLPFC activation despite no statistically significant group differences in the Stroop behavioral indices. This brain–behavior dissociation suggests that the absence of detectable differences in mean behavioral performance does not necessarily imply intact neural processing. According to the neural efficiency hypothesis, behavioral performance and neural activation may not always change in parallel, particularly when task difficulty is low to moderate (43). Under such conditions, similar mean task performance may be accompanied by differences in the amount or efficiency of neural resources recruited to support that performance.

In the present clinical context, however, reduced DLPFC activation should not be interpreted as evidence of enhanced neural efficiency. Given the established adverse effects of chronic METH exposure on prefrontal function, the observed hypoactivation may instead reflect insufficient task-related recruitment or reduced neural reserve. Thus, neural dysfunction may be detectable even when group differences are not evident in conventional behavioral indices. Moreover, because the present behavioral analyses were based on mean RT, accuracy, and DRT, the absence of significant group differences does not exclude subtle attentional fluctuations or response instability that might be captured by trial-level measures such as SDRT or CVRT.

This interpretation may also help explain inconsistencies in previous behavioral findings. Some studies have reported neural abnormalities without significant behavioral impairment (44, 45), whereas others have reported significant behavioral impairments (46–48). These discrepancies may be attributable to differences in abstinence duration, addiction severity, task difficulty, and the behavioral measures examined. Studies reporting behavioral deficits often involved individuals with shorter abstinence periods or used more demanding cognitive paradigms. Similarly, Wang et al (49) reported neural abnormalities in individuals with severe internet gaming disorder in the absence of significant behavioral differences, supporting the possibility that neural dysfunction may be detectable independently of deficits in mean behavioral measures. Clinical heterogeneity may further contribute to variation in task-related neural and behavioral findings across METH samples (50).

### 4.2 Moderating role of abstinence duration

The absence of significant behavioral differences between the overall SMUD and HC groups may partly reflect heterogeneity across abstinence stages. When individuals at different stages of abstinence are analyzed as a single group, stage-related differences in behavioral performance and neural–behavioral relationships may be obscured. Consistent with this possibility, abstinence duration significantly moderated the association between CH43 activation and DRT, indicating that the relationship between DLPFC recruitment and the RT Stroop difference score varied across abstinence duration.

Importantly, this moderation effect did not reflect a direct linear relationship between abstinence duration and the absolute magnitude of CH43 activation. Abstinence duration was not significantly correlated with CH43 activation across SMUD participants (r = 0.091, p = 0.442), and CH43 activation did not differ significantly between the STW and LTW subgroups. These findings suggest that abstinence duration may influence how DLPFC activation is related to behavioral performance rather than simply increasing or decreasing the magnitude of DLPFC activation.

At shorter abstinence duration, greater CH43 activation was associated with a larger DRT (B = 1137.10, SE = 434.09, p = 0.011). This pattern indicates that greater DLPFC recruitment was not accompanied by a more favorable RT difference score. From a neural-efficiency perspective, such a pattern is consistent with inefficient or compensatory recruitment of prefrontal resources, because greater neural engagement did not translate into better behavioral performance. At the mean abstinence duration, this association was attenuated and only marginally significant (B = 443.38, SE = 258.51, p = 0.092), whereas at longer abstinence duration, the association was negative but nonsignificant (B = −250.33, SE = 299.23, p = 0.407). Thus, the functional relationship between DLPFC activation and behavioral performance appeared to vary across abstinence duration.

The subgroup analyses provided complementary evidence. CH43 activation was significantly correlated with DRT in the STW subgroup (r = 0.423, p = 0.011), but not in the LTW subgroup (r = −0.017, p = 0.918). However, the direct comparison between the two correlation coefficients did not reach conventional statistical significance (Fisher z = 1.915, p = 0.055). Therefore, although the subgroup pattern was consistent with the continuous moderation analysis, evidence that CH43–DRT coupling was stronger in STW than in LTW remains suggestive rather than conclusive.

The STW subgroup also showed a larger DRT than the LTW subgroup (51). However, this difference was accompanied by shorter coRT rather than a significant difference in incoRT or accuracy. It should therefore not be interpreted as definitive evidence of greater conflict-specific slowing or a purely inhibitory-control deficit. Instead, DRT may reflect Stroop-related interference together with differences in general processing speed or baseline reaction time. Accordingly, the neural-efficiency interpretation is based primarily on the abstinence-dependent CH43–DRT relationship, rather than on the subgroup difference in DRT alone.

Taken together, these findings suggest that early abstinence may be characterized by less efficient DLPFC activation–behavior coupling, whereby greater prefrontal recruitment is associated with a larger RT difference score. With longer abstinence, this inefficient coupling may become attenuated or reorganized. This interpretation is consistent with evidence that early abstinence is associated with frontostriatal dysregulation, dopaminergic disruption, and increased cortical demands (19), whereas prolonged abstinence has been associated with partial recovery of prefrontal network function and behavioral performance (52). Nevertheless, because the present study used a cross-sectional design, the observed abstinence-related pattern cannot establish within-person neural recovery. Longitudinal studies are required to determine whether it reflects recovery, functional reorganization, or differences between participant cohorts.

### 4.3 Activation-behavior association in short-term abstinence

The stepwise regression analysis in the STW subgroup further supports the presence of inefficient activation–behavior coupling during early abstinence. Higher left DLPFC activation was associated with longer DRT, indicating that greater prefrontal engagement was not accompanied by better behavioral performance. This “higher activation, poorer performance” pattern suggests maladaptive or inefficient recruitment rather than functional recovery.

Similar patterns have been reported in other substance use disorders, including opioid and cocaine use disorders, in which early abstinence is often associated with inefficient recruitment of prefrontal control regions (52, 53). In SMUDs, Chen et al. (54) found that intermittent theta burst stimulation over the left DLPFC reduced β-wave oscillations and improved addiction Stroop performance in patients with less than 4.5 months of abstinence, supporting the relevance of DLPFC dysfunction during early abstinence. Other studies have reported increased prefrontal gray matter volume after several months of abstinence, potentially related to brain-derived neurotrophic factor-mediated neuroplasticity (53). Elevated frontal glutamine levels after 1–2 months of abstinence may also reflect a compensatory metabolic response that evolves across abstinence stages (19).

Together, these findings suggest that early abstinence represents a period of marked neural dysregulation, during which DLPFC recruitment may be insufficient or inefficiently coupled with behavioral control. Therefore, the early abstinence period may constitute a critical window for interventions targeting DLPFC function, with the potential to improve inhibitory control and clinical outcomes (55, 56).

### 4.4 Limitations

Several limitations of this study should be acknowledged. Firstly, the restricted spatial resolution of fNIRS limits signal acquisition to superficial cortical regions, thereby precluding the assessment of subcortical structures such as the striatum and amygdala, which are crucial components of addiction-related neurocircuitry. Secondly, the cross-sectional design of the study restricts the ability to infer temporal changes in activation-behavior coupling, underscoring the necessity for longitudinal studies to elucidate recovery trajectories. Thirdly, while the restriction of the sample to male participants minimizes sex-related variability, it concurrently limits the generalizability of the findings, especially in light of emerging evidence indicating sex-specific neural adaptations in MUD (57). Fourthly, the present behavioral analyses were based on conventional Stroop indices, including coACC, coRT, incoACC, incoRT, and DRT, and did not examine intra-individual RT variability. Therefore, the absence of significant group differences in these indices does not exclude subtle attentional fluctuations or response instability in SMUDs. Trial-level measures such as SDRT and CVRT may provide complementary information beyond mean RT and accuracy. Future studies should incorporate a separately specified trial-level preprocessing and RT-variability analysis framework. Lastly, because the classic color-word Stroop task may have limited sensitivity to subtle inhibitory-control abnormalities, future work should incorporate more sensitive cognitive-control paradigms and multidimensional cognitive assessments.

## 5 Conclusions

In summary, this study presents new evidence that abstinent individuals with SMUD exhibit hypoactivation of the left DLPFC during inhibitory control processing. This finding was further supported by covariate-adjusted sensitivity analyses controlling for years of education and AUDIT scores. In addition, the relationship between DLPFC activation and Stroop interference was moderated by METH abstinence duration. Specifically, early abstinence may be characterized by less efficient activation–behavior coupling, pointing to this period as a critical window for interventions aimed at enhancing inhibitory control. These insights contribute to a deeper understanding of the neural mechanisms underlying recovery in SMUD and highlight the necessity of targeted neuromodulatory strategies to improve clinical outcomes.

## Data Availability

The raw data supporting the conclusions of this article will be made available by the authors, without undue reservation.
