# Supplementary material for: Abnormal DLPFC activation in severe methamphetamine use disorder during abstinence: fNIRS evidence from Stroop task
Source: Front Psychiatry. 2026 Aug 5;17:1863806. doi: 10.3389/fpsyt.2026.1863806 (PMC13488168; doi:10.3389/fpsyt.2026.1863806)
Supplement: Supplementary file 1 [file Table1.docx]

**Table S1. Covariate-adjusted analysis of significant fNIRS channels.**

| Variables | *ANCOVA F for group* | *p value* | *Partial η²* | Covariates |
| --- | --- | --- | --- | --- |
| CH32 (mmol/L) | 18.2142 | <0.0001 | 0.1347 | Education, AUDIT |
| CH43 (mmol/L) | 13.9294 | 0.0003 | 0.1064 | Education, AUDIT |

Note: CH32/43, HbO activation in channel 32 and 43.
